# Trends of Antibiotic Resistance in ESKAPE Pathogens in Mbarara Regional Referral Hospital (2015–2022), South Western, Uganda

**DOI:** 10.1155/cjid/7034931

**Published:** 2025-05-14

**Authors:** Joel Bazira, Pauline Petra Nalumaga, Balukhu Quraishi, Abel W. Walekhwa, Mugisha Lawrence, Jacob Stanley Iramiot

**Affiliations:** ^1^Department of Microbiology, Mbarara University of Science and Technology, Mbarara, Uganda; ^2^Department of Veterinary Medicine, University of Cambridge, Cambridge, UK; ^3^Department of Veterinary Medicine, Makerere University, Kampala, Uganda; ^4^Department of Microbiology, Busitema University, Tororo, Uganda

**Keywords:** antimicrobial resistance, ESKAPE pathogens, southwestern Uganda

## Abstract

**Introduction:** Antimicrobial resistance remains a global threat, with increasing infection and death rates. The World Health Organization identified *Enterococcus faecium*, *Staphylococcus aureus*, *Klebsiella pneumoniae*, *Acinetobacter baumannii*, *Pseudomonas aeruginosa*, and *Enterobacter* spp. (ESKAPE) as priority pathogens due to their increased antibiotic resistance development. This study assessed the resistance patterns of ESKAPE pathogens from 2015 to 2022 in Mbarara Regional Referral Hospital, Uganda.

**Methods:** A retrospective study was conducted by reviewing retrieved data from WHONET. This is the laboratory software used in the microbiology laboratory in the Department of Microbiology, Mbarara University of Science and Technology (MUST), which receives samples from both the outpatient and the inpatient departments of Mbarara Regional Referral Hospital.

**Results:** A total of 5733 bacterial isolates were recovered, of which, 4822 were ESKAPE pathogens from the collected clinical specimens including blood, stool, urine, swabs, cerebral spinal fluid, wounds, and sputum. *Staphylococcus aureus* (4291, 74.8%) was the most frequently isolated pathogen followed by *Klebsiella pneumoniae* (345, 6.0%). The bacteria categorized as ESKAPE pathogens showed significant rates of multidrug resistance. Ampicillin showed the highest resistance followed by ciprofloxacin.

**Conclusion:** The significant prevalence of antimicrobial resistance to penicillin, ciprofloxacin, and tetracycline in ESKAPE bacteria emphasizes the significance of enhancing antimicrobial surveillance and infection-prevention and management initiatives within the country.

## 1. Introduction

The increasing prevalence of multidrug-resistant bacteria [1, 2] has been associated with a decrease in antibiotic development [3]. Antimicrobial-resistant (AMR) bacteria are classified as a serious threat to human health by both the World Health Organization (WHO) and the United States Centers for Disease Control and Prevention (CDC) [4, 5]. AMR is caused by increased selection pressure from excessive use of antibiotics, which leads to bacterial adaptation mechanisms. Although there is currently no organized international surveillance of AMR (3), available data suggest AMR infections that are acquired in hospitals and the community result in about 33,000 mortality rate each year in Europe, costing the continent $1.5 billion [6, 7]. Infectious diseases remain the largest cause of death, a condition worsened by emerging and reemerging infectious diseases in developing countries such as Uganda, where economic loss figures are unknown [8–10].

In February 2017, the WHO published a list of pathogenic organisms in order to focus on intensive research and urgent new drug development. *Enterococcus faecium, Staphylococcus aureus, Klebsiella pneumoniae, Acinetobacter baumannii, Pseudomonas aeruginosa, and Enterobacter species* (ESKAPE) pathogens [11] were given “priority status” [12] within this broad list. ESKAPE pathogens have developed antibiotic resistance through genetic variations and the acquisition of mobile genetic elements (MGEs) [13] to common antibiotics such as carbapenems, glycopeptides, polymyxins, lipopeptides, macrolides, fluoroquinolones, tetracyclines (TETs), and lipoglycopeptides [14]. The ESKAPE group of bacteria with these drug resistance mechanisms have increased in hospital and community-acquired infections [15, 16] presenting clinicians with serious treatment challenges [17]. This has resulted in high mortality and morbidity rates and increased healthcare costs [18].

In Uganda, a structured national AMR surveillance program in alignment with the WHO Global AMR Surveillance System (GLASS) is implemented. The available AST data indicate high resistance to the recommended and prescribed antibiotics for infection therapy [19]. This study examined trends in the annual antibiotic resistance rates for all ESKAPE pathogens analyzed from 2015 to 2022 from samples received from patients attending Mbarara Regional Referral Hospital (MRRH), Uganda.

### 1.1. Significance of this Study

Effective antibiotic prescription and controlling the spread of resistant bacteria within a particular region are made possible by the systematic collection of AMR data at the local, regional, and national levels. This allows a wide range of high-quality data to be comparable and shared in global networks. A resistance surveillance dataset from the GLASS in Uganda from 2015 to 2020 [19] indicated AMR trends from hospital isolates (urine, blood, stool, and urogenital swabs). However, there are no research findings of long-term surveillance data describing the antibiotic resistance patterns of ESKAPE infections in the Mbarara district in Uganda. Therefore, the purpose of this study is to describe the trends of antibiotic resistance among ESKAPE pathogens during an 8-year period from samples received from patients attending MRRH, Uganda. This surveillance study may provide reliable data for empiric therapy in order to reduce the infection rate associated with ESKAPE bacteria in the western region of Uganda.

## 2. Methods

This was a retrospective study that examined annual data from 2015 to 2022 from the microbiology laboratory of the Department of Microbiology, MUST, which receives samples from both the outpatient and inpatient departments of MRRH. MRRH is located in Mbarara, western Uganda, about 260 km from Kampala capital city, serving as a teaching hospital for Mbarara University of Science and Technology (MUST) and several institutions [20]. The hospital has over 300 beds and provides a wide range of health services to the western region and neighboring countries such as Tanzania and Rwanda through its departments which include pediatrics, obstetrics and gynecology, internal medicine, surgery, cancer unit, emergency and critical care, imaging, pathology, and laboratories [21, 22].

This study evaluated data from antibiotic susceptibility results of bacteria isolated from clinical specimens collected from patients for diagnosis. After growth on blood agar and MacConkey agar, the isolated bacteria were identified using biochemical tests of the isolated colonies [23]. The Kirby–Bauer disk diffusion method was used for antibiotic susceptibility and results are interpreted according to the Clinical and Laboratory Standards Institute (CLSI) standards [24]. Multidrug resistance was defined as being resistant to at least one agent from at least three antimicrobial groups [1]. The resistance analysis was focused on the ESKAPE pathogens which are monitored globally and regionally within the surveillance networks [15]. The antibiotics examined included penicillin (ampicillin [AMP]), fluoroquinolones (ciprofloxacin [CIP]), aminoglycosides (gentamicin [GM]), and TETs (TET), and chloramphenicol because they are readily available in our setting [25]. The control strains used in this microbiology laboratory were S*. aureus* ATCC 25923 and *Klebsiella pneumoniae* ATCC 700603 [26].

### 2.1. Data Extraction and Analysis

All information about specimen type, bacterial isolation, and susceptibility pattern is deposited on the WHONET system, a computerized laboratory database restricted to only laboratory staff working in the department of microbiology, MUST. Patient confidentially was maintained at all times. Isolate information, specimen type, and results of antimicrobial susceptibility testing were extracted. Once the data were extracted, trends in the total number of ESKAPE pathogens and their AMR patterns were determined. These trends were compared over a 7-year period. Data analysis was performed using Stata 17.0 software SE (StataCorp. LP. 2013, College Station, Texas, United States). The categorical data was presented using stratified frequency tables (numbers and percentages). Trends or associations were assessed using the standard Pearson's chi-square [26].

## 3. Results

### 3.1. Distribution of Species by Clinical Specimens

For the period 2015–2022, a total of 5733 bacterial isolates were recovered from clinical specimens and 4822 (84.1%) were ESKAPE pathogens. The clinical specimens included blood, stool, urine, swabs, cerebral spinal fluid, wounds, and sputum. Overall, *S. aureus* (4291, 74.8%) was the most frequently isolated pathogen followed by *K*. *pneumoniae* (345, 6.0%), *P. aeruginosa* (67, 1.2%), *Enterococcus faecium* (66, 1.1%), *Enterobacter* spp. (47, 0.8%) and *Acinetobacter* spp. was the least isolated bacteria (6, 0.1%) as shown in Table 1.

### 3.2. Antibiotic Resistance

During the study, the ESKAPE infections exhibited substantial levels of antibiotic resistance. Overall, the most prevalent resistance pattern among Gram-negative ESKAPE pathogens was highly resistant to AMP, with *Klebsiella pneumoniae* expressing the highest frequency. *Staphylococcus aureus* also showed increased resistance to AMP and TET but moderate resistance to gentamycin and chloramphenicol.

**Figure 1:**
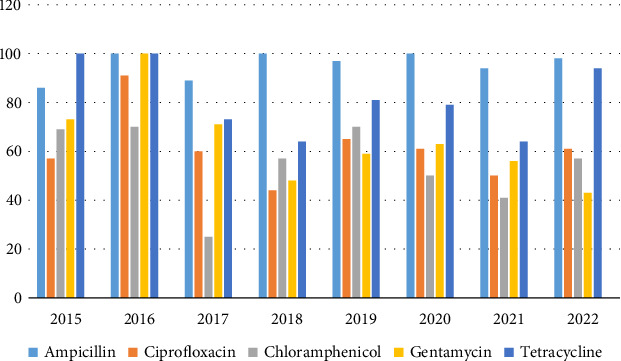
Resistance patterns of *Klebsiella pneumoniae* (2015–2022) in percentage. *K. pneumoniae* showed average resistance to ciprofloxacin, that is, 57%, which increased to 61% in 2022. Fluctuating average resistance to chloramphenicol, gentamycin, and tetracycline was observed from 69%, 73%, and 100% in 2015 to 57%, 43%, and 94% in 2022, respectively.

Between 2015 and 2022, 345 *K. pneumoniae* isolates were recovered. Eight isolates were resistant to chloramphenicol and 92 isolates were resistant to TET, while 176 isolates were resistant to CIP. Increasing AMR trends were observed in *K. pneumoniae* (Figure 1).

**Figure 2:**
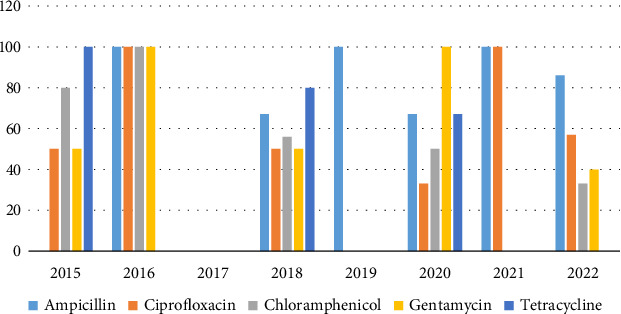
Resistance trends of *Enterobacter* species (2015–2022) in percentage. Between 2015 and 2022, *Enterobacter* species exhibited increased resistance to ciprofloxacin from 50% to 57% in 2022. Resistance to ampicillin averagely fluctuated from 100% in 2016 to 86% in 2022. Decreasing resistance trends across gentamicin (50%–40%), chloramphenicol (80%–33%), and tetracycline (100%–67%) were observed.

Between 2015 and 2022, 47 *Enterobacter* spp. isolates were recovered. 13 *Enterobacter* spp. isolates were resistant to AMP, 17 isolates were resistant to chloramphenicol, and 14 isolates were resistant to TET, while 16 isolates were resistant to CIP. Increasing AMR trends were observed in *Enterobacter* spp. especially toward CIP (Figure 2).

**Figure 3:**
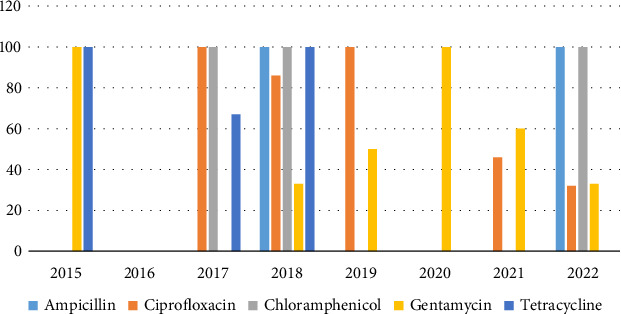
Resistance trends of *Pseudomonas aeruginosa* (2015–2022). Accumulative average resistance to ciprofloxacin (25%–32%) and decreased resistance to gentamycin (100%–33%) were observed in *P. aeruginosa*.

Between 2015 and 2022, 67 isolates of *Pseudomonas aeruginosa* were isolated. 31 isolates were resistant to AMP, 37 isolates were resistant to CIP, 20 isolates were resistant to chloramphenicol, 37 isolates were resistant to gentamycin, and 29 isolates were resistant to TET. Over the study period, an increase in the antibiotic resistance in *P. aeruginosa* was noted particularly to chloramphenicol, TET, and AMP (Figure 3).

**Figure 4:**
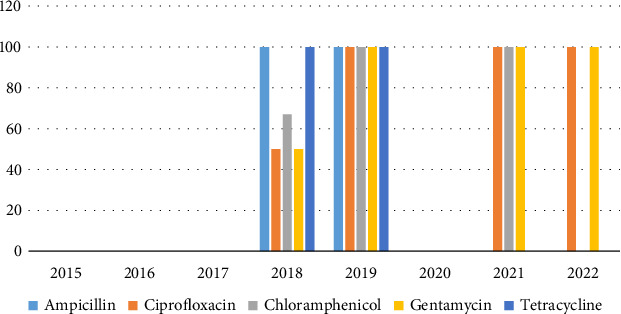
Resistance trends of *Acinetobacter baumannii* (2018–2022).

**Figure 5:**
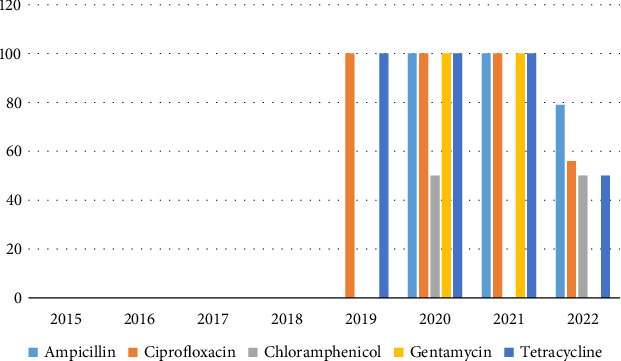
Resistance pattern of *Enterococcus* species (2019–2022).

High multidrug resistance rates were observed for *Acinetobacter baumannii* and *Enterococcus* spp. with over 50% of isolates resistant to AMP, CIP, chloramphenicol, gentamycin, and TET (Figures 4 and 5, respectively).

**Figure 6:**
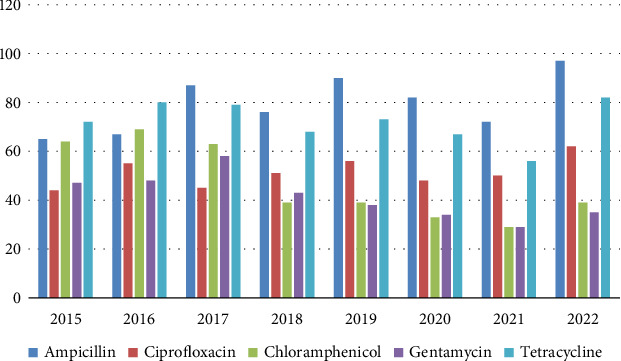
Resistance patterns of *Staphylococcus aureus*. A decreasing trend in the proportion of resistant isolates of *S. aureus* in chloramphenicol and gentamycin from 64% and 47% to 39% and 35%, respectively, and increased resistant isolates to ampicillin, ciprofloxacin, and tetracycline from 65%, 44%, and 72% to 97%, 62%, and 82%, respectively.

4291 isolates of *S. aureus* were isolated over the study period, of which, 336 were resistant to AMP, 596 were resistant to CIP, 380 were resistant to chloramphenicol, and 400 were resistant to gentamycin, while 488 were resistant to TET. As illustrated in Figure 6, there were varying rates of resistance to *Staphylococcus aureus*.

## 4. Discussion

According to this study, the ESKAPE pathogens made the majority of the isolated organisms in the samples received in the microbiology department from 2015 to 2022, with an average increase in penicillin resistance. AMP, a commonly used penicillin-class antibiotic, exhibited the highest resistance rates, particularly among *Staphylococcus aureus* and *Klebsiella pneumoniae*. These findings align with national surveillance data from Uganda, which also report high resistance patterns to penicillins [27]. The frequent prescription of AMP in Uganda's integrated management of various infections, including sepsis, pneumonia, and urinary tract infections, may have contributed to an increase in penicillin-resistant bacteria [28]. Similar trends have been observed in studies across Africa, for example, in Kenya [29], South Africa [30], and other parts of the world [31–33], suggesting a widespread issue of penicillin resistance across different regions.

Increasing trends of resistance rate to CIP against all the organisms were high, especially among *Klebsiella pneumoniae* and S. *aureus*. This is concerning given that CIP is a key antibiotic in Uganda's treatment guidelines, commonly prescribed for gastrointestinal infections and as a second-line therapy for uncomplicated UTIs. These results are comparable to a study carried out in 2015 in Mulago [34]. CIP resistance in *K. pneumoniae* and *Acinetobacter baumannii* is due to mutations in the genes gyrA and parC reducing affinity to CIP [35]. The increasing CIP resistance among these pathogens may also be due to the increased use of CIP for the treatment of penicillin-resistant bacteria, reinforcing the need for antibiotic stewardship programs. Therefore, the observed increasing rates of resistance to CIP over time correlate with the literature, which states that the increased use of antibiotics leads to increased bacterial resistance [36].

Chloramphenicol resistance displayed mixed trends. A general decline was observed across the pathogens except *Enterococcus* spp. and *Pseudomonas aeruginosa.* This can be explained by the limited use of chloramphenicol globally [37], despite being a very inexpensive broad-spectrum antibiotic. In comparison, a study in Israel [38] had similar results and attributed the reduced resistance to chloramphenicol therapy to its replacement by fluoroquinolones, which are highly effective against both Gram-negative and Gram-positive bacteria [39]. The significantly increased resistance exhibited by *Pseudomonas aeruginosa* to chloramphenicol could be attributed to intrinsic resistance [40] due to its outer membrane, which has very low nonspecific permeability to tiny hydrophilic compounds [41] and multidrug efflux systems based on resistance-nodulation division (RND) [42]. Increased resistance shown by *Enterococcus* spp. may be due to the efflux of free-drug molecules that release bound chloramphenicol from the ribosome [43].

There was a noticeable decrease in gentamycin resistance in most pathogens except *Acinetobacter* spp. Aminoglycosides such as gentamycin are primary treatment options for *Acinetobacter*-related infections, but resistance is commonly mediated by aminoglycoside-modifying enzymes which inactivate the antibiotics [44]. Studies from Iran and Western Uganda have reported similar high gentamycin resistance rates in *Acinetobacter baumannii* (up to 86%) [8, 44]. The persistence of gentamycin resistance in *Acinetobacter baumannii* highlights the need for alternative therapies and improved infection control strategies.

The increasing resistance to commonly used antibiotics in ESKAPE pathogens emphasizes the urgent need for antimicrobial stewardship. In Uganda, inappropriate antibiotic prescriptions, self-medication, and the availability of over-the-counter antibiotics contribute significantly to resistance [8]. The increasing trends of antibiotic resistance found in this review suggest that the commonly used and existing antibiotics may be rendered noneffective without quick action. This calls for antimicrobial stewardship procedures and the development of new antibiotics.

AMR is rapidly increasing globally including in Uganda [45], threatening every person's health, economic, and social status [46]. This is the first study to describe resistance trends among WHO global priority pathogens in Uganda. However, standard treatment guidelines from the Ministry of Health of Uganda and local prescribing patterns influenced the antibiotic selection in this study.

## 5. Conclusion

The findings of the research reveal an increase in resistance to routinely used antibiotics such as CIP and penicillins among ESKAPE bacteria between 2015 and 2022. This rise highlights the growing issue of AMR, particularly among clinically relevant bacteria. The findings point to a potential decline in the efficacy of routinely used antibiotics, which could have major implications for the treatment of ESKAPE infections. This pattern demands urgent responses, such as the establishment of effective antimicrobial stewardship programs, increased resistance surveillance, and the development of novel therapeutic techniques. Public health experts, researchers, and healthcare professionals must collaborate together to solve the effects of AMR and preserve the efficacy of currently available antibiotics.

## Figures and Tables

**Table 1:** The distribution of ESKAPE pathogens in clinical specimens received.

ESKAPE pathogens	Blood		Pus swab		Urine		High vaginal swab		CSF		Nasal swab		Stool		Urethral swab		Ear swab		Sputum	
	*N*	%	*N*	%	*N*	%	*N*	%	*N*	%	*N*	%	*N*	%	*N*	%	*N*	%	*N*	%
*Enterobacter* spp.	7	0.4	15	1	8	0.8	3	0.5	13	3.5	—	—	1	0.5	—	—	—	—	—	—
*Pseudomonas aeruginosa*	1	0.07	30	2	21	2.2	2	0.4	4	1	—	—	1	0.5	1	1.6	6	3.7	1	0.7
*Staphylococcus aureus*	1409	91.6	1150	77.3	538	57.6	444	83.6	194	51.5	266	87.2	32	15.9	49	79	146	91.8	63	45.3
*K. pneumoniae*	7	0.4	133	8.9	154	16.5	33	6.2	3	0.8	1	0.3	2	0.9	1	1.6	2	1.2	9	6.5
*Acinetobacter baumannii*	—	—	2	0.1	3	0.3	1	0.2	—	—	—	—	—	—	—	—	—	—	—	—
*Enterococcus faecium*	7	0.4	15	1	8	0.9	3	0.5	13	3.4	—	—	1	0.4	—	—	—	—	—	—
*Other bacteria*																				
*Proteus mirabilis*	—	—	30	2	7	0.7	—	—	2	0.5	—	—	—	—	—	—	—	—	—	—
*Salmonella* spp.	62	4	—	—	—	—	—	—			—	—	12	6	—	—	—	—	—	—
*Neisseria gonorrhoeae*	—	—	—	—	19	2	—	—			—	—	—	—	7	11.2	—	—	—	—
*Vibrio cholerae*	1	0.07	—	—	—	—	—	—			—	—	152	75.6	—	—	—	—	—	—
*Citrobacter* spp.	4	0.3	88	5.9	164	17.6	38	7.1	2	0.5	—	—	—	—	3	4.8	1	0.6	7	5
*Streptococcus* spp.	40	2.6	25	1.7	12	1.2	7	1.3	145	38.6	38	12.4	—	—	1	1.6	4	2.5	59	42.4

Total	1538		1488		934		531		376		305		201		62		159		139	

## Data Availability

All datasets generated during this research are accessible upon request from the corresponding author and can be shared in accordance with Mbarara University of Science and Technology's data sharing act.
